# The vocal organ of hummingbirds shows convergence with songbirds

**DOI:** 10.1038/s41598-020-58843-5

**Published:** 2020-02-06

**Authors:** Tobias Riede, Christopher R. Olson

**Affiliations:** Department of Physiology, College of Graduate Studies, Midwestern University, 19555 N 59th Ave, Glendale, AZ 85308 United States

**Keywords:** Evolution, Animal behaviour, Animal physiology, Biomechanics

## Abstract

How sound is generated in the hummingbird syrinx is largely unknown despite their complex vocal behavior. To fill this gap, syrinx anatomy of four North American hummingbird species were investigated by histological dissection and contrast-enhanced microCT imaging, as well as measurement of vocalizations in a heliox atmosphere. The placement of the hummingbird syrinx is uniquely located in the neck rather than inside the thorax as in other birds, while the internal structure is bipartite with songbird-like anatomical features, including multiple pairs of intrinsic muscles, a robust tympanum and several accessory cartilages. Lateral labia and medial tympaniform membranes consist of an extracellular matrix containing hyaluronic acid, collagen fibers, but few elastic fibers. Their upper vocal tract, including the trachea, is shorter than predicted for their body size. There are between-species differences in syrinx measurements, despite similar overall morphology. In heliox, fundamental frequency is unchanged while upper-harmonic spectral content decrease in amplitude, indicating that syringeal sounds are produced by airflow-induced labia and membrane vibration. Our findings predict that hummingbirds have fine control of labia and membrane position in the syrinx; adaptations that set them apart from closely related swifts, yet shows convergence in their vocal organs with those of oscines.

## Introduction

Due to their small body size, hummingbirds have experienced selection for a number of traits that have set them apart from other avian lineages^1^. Various modes of acoustic communication are among those traits^2^. For example, some hummingbirds use elements of their plumage to generate sounds for effective communication with conspecifics^3^. Like other birds, hummingbirds also produce a diverse and complex vocal repertoire^4–6^ whose neural control mechanisms suggest convergence to those of the distantly related songbird lineage^7,8^. Despite their vocal complexity and similarity of their vocal learning with songbirds, vocal production mechanisms of the hummingbird syringeal sound source are poorly understood.

Acoustic communication requires a sound production mechanism that is congruent with a species’ hearing ability, acoustic environment and physical capability^9^. The occurrence of vocalizations with exceptionally high fundamental frequency (F_0_) in some hummingbirds^10–12^ reveals that the hummingbird lineage has vocal abilities that occur outside those of other avian taxa. The hummingbird syrinx possesses a more complex anatomy than closely related taxa such as swifts or oilbirds^13^. One adaptation that sets hummingbirds apart from other avian species is the extrathoracic placement of their syrinx in the neck region rather than in the thorax^13–18^. Previous morphological studies of the hummingbird syrinx have also described the presence of a calcified tympanum, a certain number of accessory cartilages, one or two pairs of intrinsic muscles and the tracheolateralis muscle^13–17,19^. Investigations of the syringeal functional morphology have always been challenged by the organ’s small size and simultaneous complexity^20–25^, but the recent use of contrast-enhanced microCT imaging allows great progress in describing these acoustic organs^26–28^.

**Figure 1:**
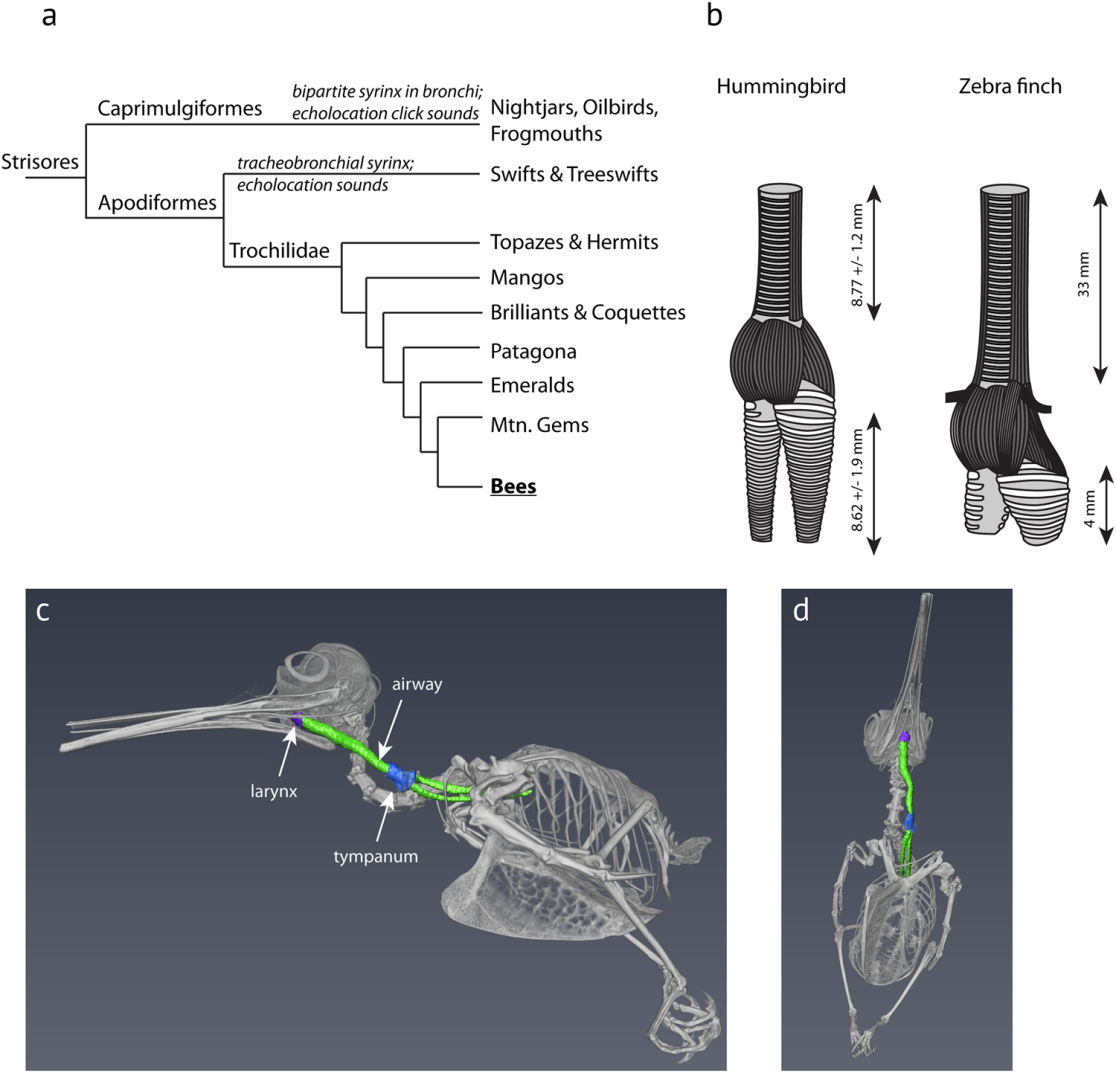
(**a**) The “bee” hummingbirds (Mellisugini) have undergone rapid speciation relative to related groups^1^. Hummingbirds(Trochilidae) along with swifts and swiftlets are grouped within the Apodiformes, while Caprimulgiformes and Apodiformes are within the clade Strisores. (**b**) The hummingbird vocal organ is located in the neck, unlike in other birds where it is located in the body cavity. The hummingbird tracheobronchial junction is positioned much higher in the respiratory tract than most other avian vocal organs. Consequently, the tracheal (i.e. the tracheal vocal tract filter) is much shorter than for example that of a zebra finch, a relatively small songbird. Tracheal and bronchial measurements of hummingbirds represent mean values of four similar species (mean ± standard deviation). Zebra finch dimensions after Daley & Goller^67^. Zebra finch syrinx schematic after Riede & Goller^24^. (**c**) Lateral view of a segmented 3D surface of the skeleton (white), the tracheal and bronchial airway (green), the tympanum (blue) and the larynx (purple) of an Anna’s hummingbird adult male that was microCT scanned at 50 μm resolution in a fixed position. (**d**) Ventral view of the same bird. Note that the calcified tympanum of the syrinx is located on the bird’s left side of the neck. The bird was salvaged after a window collision which resulted in the rostral part of the beak being lost.

Our initial goal was to explore the anatomy and function of the smallest avian sound source and evaluate previous statements about its similarity to the passerine syrinx. This was carried out by examination of vocal organs using contrast-enhanced microCT imaging informed by histology. We also let hummingbirds vocalize in a heliox environment to experimentally investigate their sound production mechanism. We utilized four species of free-living “bee” hummingbirds (tribe: Mellisugini) that occur in the SW United States. This clade has undergone a high rate of speciation compared to other clades (Fig. 1a)^1^, and all demonstrate species-specific multi-modal courtship behavior, including differing degrees of plumage-based visual signaling, production of species-specific feather sounds, as well as differences in vocal song complexity including learned song in two species and the lack of song in two others^2,29^. Thus, we were also curious how the differing degrees of vocal complexity that is found among these very closely related species may be reflected in their peripheral vocal organs. Here we show that recent speciation includes divergence in the anatomy of their vocal organs.

## Methods

### Animals

We investigated syrinx histology and morphology in Anna’s hummingbirds (ANHU, *Calypte anna*), Costa’s hummingbirds (COHU, *C. costae*), rufous hummingbirds (RUHU, *Selasphorus rufus*), and black-chinned hummingbirds (BCHU, *Archilochus alexandri*) for a total of 18 hummingbirds. A summary of species and sex is provided in Supplementary Table 1. A single male ANHU was salvaged immediately following a window collision and used for whole-body microCT imaging (Fig. 1c,d), and a separate group of ANHUs were used for vocal recording (N = 3) and not used for histological or morphological comparisons. We captured all four species in Cochise, Pima, Maricopa, Gila and Coconino Counties (Arizona) during the months of June-August of 2016–2017 under permits from the Arizona Department of Game and Fish and the US Fish and Wildlife Service. All procedures were approved by the Institutional Animal Care and Use Committee at Midwestern University, Glendale, AZ, USA (IACUC Nos. 2818 and 2892) following established guidelines for use of wild birds in research^30^.

### Histology of the vocal organ

Animals were euthanized with isoflurane and exsanguinated by transcardial perfusion with 0.9% saline, chased with 4% paraformaldehyde in PBS. We measured lengths of trachea and bronchi *in situ*. Syrinxes were then dissected and fixed in 10% buffered formalin phosphate (SF100-4; Fisher Scientific) for one week. Syrinxes were paraffin embedded and sectioned at 5 μm tissue thickness in the frontal plane from ventral to dorsal, retaining samples at 50 μm intervals. Samples were investigated for the presence of collagen, elastin and hyaluronan, all of which play an important role in biomechanics of vocal fold vibration^25^. Sections were stained with haematoxylin–eosin for a general overview, Masson’s Trichrome for collagen fiber stain, Elastica–Van Gieson for elastic fiber stain or Alcian blue (AB) stain (pH 2.5) for mucopolysaccharides and glycosaminoglycans. We also performed a digestion procedure with bovine testicular hyaluronidase (2 h at 37 °C) in combination with a subsequent AB stain. Incubation with bovine testicular hyaluronidase increases specificity for various acid mucosubstances in the AB stain. If hyaluronan is a major component of the mucosubstances, AB stain fully degrades. Sections were scanned with an Aperio CS 2 slide scanner and processed with IMAGESCOPE software (v. 8.2.5.1263; Aperio Technologies, Inc.), or imaged with a Leica DM4000 uptight microscope yoked to a computer running LAS X software.

### MicroCT imaging of the syrinx

The syrinx of a male ANHU, a male and female COHU, and a male RUHU were imaged. The syrinx was excised as explained above and stained with iodine solution (0.4 g iodine/200 ml 99% ethanol) for 10 days^28,31^. Stained specimens were placed in a custom-made acrylic tube and scanned in air with 59 kV source voltage and 167 µA intensity using a Skyscan 1172 (Bruker-microCT, Kontich, Belgium). Projection images were recorded with an angular increment of 0.4° over a 180° rotation. Voxel size in the reconstructed volumes was 5.03 µm per pixel. Reconstructed image stacks were then imported into AVIZO software (FEI, version Lite 9.0.1).

Syringeal cartilages, musculature, labia and membranes and the border between the airway and soft tissues of the syrinx in the CT scans were traced manually. This approach provided outlines for the cartilaginous framework, soft tissues and for the airway. Derived 3D surfaces (STL format) and a video animation of an adult male ANHU has been archived and are available from Morphobank^32^, project # 3269.

To evaluate differences in syrinx morphology among the four hummingbird species we acquired repeatable linear measurements from all individuals using images taken of histological sections in the frontal plane under the microscope and measured with ImageJ Fiji, ver. 2.0^33^ (N = 14 syrinxes) or 3-dimensional CT-scan reconstructions (N = 4 syrinxes). These measurements were acquired by examining multiple serial sections along the z-plane and selecting the level which presented the largest measurement for each character, separately, thus the data used in the analysis are maximum length (e.g. greatest trachea diameter) available for each individual. For the four bilateral characters, we measured both sides and took the average of the right and left sides for the analysis of species differences. In some specimens these characters were also measured from the 3-D microCT-scans that were acquired by rotation of the syrinx to the plane of maximum length for each character and taking the linear measurement in AVIZO software. The two methods of measurement delivered similar results for different specimens of the same species, therefore all data were included in the analysis. Data are presented as means ± SE. Data analysis was performed with JMP 12.

### Vocalization in captivity and heliox experimentation

Free-living hummingbirds produce songs and a variety of calls that occur in diverse social contexts^34,35^. We studied vocal behavior in captivity of three male ANHUs (two adult and one juvenile). Animals were captured at a feeder near Payson, AZ and transferred to the aviary at Midwestern University. Birds were maintained on a natural light cycle and a constant temperature (24 °C) and provided with a nutritionally complete diet (Nekton Nektar-Plus) *ad libitum*. In order to investigate which vocal types can be recorded in captivity, the males were kept in a custom-made acrylic cage (50 × 50 × 200 cm) or in a wire-mesh cage (50 × 50 × 50 cm). Both cages were equipped with perches, a feeder and fresh water. In both cages their vocalizations were continuously monitored with a microphone (AKG, C417L; 0.02–18 kHz; or a GRAS ½“ Pressure Microphone Type 40AG with a GRAS¼” Pre- amplifier Type 26AC) placed inside the cages next to a single perch. Signals were sampled at 44.1 kHz and saved as uncompressed files on a computer using Avisoft Recorder software (Avisoft‐Bioacoustics; Glienicke, Germany). These hummingbirds shared a room with a zebra finch colony, which were audible on the recordings, but at a much-reduced amplitude.

The birds were also used in heliox experiments in order to investigate their vocal production mechanisms. If the sound production in hummingbirds follows the same principles as in other birds^36^, we expect no change in the fundamental frequency in the light gas atmosphere. Alternatively, if the sound production mechanism is an aerodynamic whistle, we expect that fundamental frequency will increase proportionally to the density change of the breathing gas. Based on previously established protocols for heliox delivery^37–39^ individual males were placed inside the acrylic cage and various call types, but not learned song, were recorded in normal air and heliox atmospheres. Heliox gas (79% He, 21% O_2_) flooded the cage through a 12 mm diameter tube placed in the cage wall at flow rates of 20–40 L·min^−1^. In light gas the fundamental frequency (F_0_) of a whistle increases in proportion to the amount of the gas present. Light gas concentrations were estimated by measuring the frequency change of a small whistle placed in the wall of the cage and connected externally by a rubber hose, where the ratio of the frequency of the whistle in air and in heliox provided an expected effect for any given heliox concentration. The whistle was blown and recorded at regular intervals to monitor the heliox concentration. Different call types produced in normal air and in heliox atmosphere were analyzed for total duration, F_0_ and relative sound pressure level. Reported means of maximum sound pressure level values are not relative to a common standard and were only compared within individuals between treatments. All measurements were performed using PRAAT sound analysis software (v. 5.3.80 for Windows; www.praat.org).

## Results

### Syrinx histology and morphology

The tracheobronchial junctions of all specimens were located in the neck, resulting in short trachea and much longer bronchi than is typical for small birds (Table 1; Fig. 1b–d). Trachea length *in situ* was 8.77 ± 1.2 mm (N = 9). Bronchi length was 8.62 ± 1.9 mm. The syrinx was located at the tracheobronchial junction and showed well-developed musculature in all species, regardless of sex. This initial description is general for all four species and sexes and is focused on (a) the cartilaginous framework of the syrinx, (b) the musculature and (c) the soft tissues most likely involved in the sound production process (i.e. two sets of lateral labia and the medial tympaniform membrane (MTM)). We then present species and sex differences based on linear measurements of the available histological specimens. A detailed species-level analysis of tissue elastic components was beyond the scope of the present study. However, three-dimensional reconstructions of cartilaginous and soft tissue elements composing a male Anna’s hummingbird syrinx can be viewed in an interactive pdf file that allows the viewer to rotate and inspect each element that is described in the following three sections (Supplementary Fig. 1).

**Table 1:** The hummingbird’s tracheal dimensions are relatively shorter than in other birds.

	Bee Hummingbirds	Zebra finch	White-rumped Swiftlet^C^	Oilbird^D^
Body mass (BM in kg)	0.0035–0.01	0.015	0.010	0.419
Trachea length (TL in cm)	0.88 ± 0.12	3.3^*^	2.2	11
Expected tracheal length^A^ (in cm) TL = 16.77 *BM^0.394^	1.8–2.7	3.2	2.72	11.9
Tracheal diameter (TD in cm)	0.09	0.12	0.15	4.4
Expected tracheal diameter^A^ (in cm) TD = 0.531*BM^0.348^	0.074–0.106	0.12	0.11	0.39

**Figure 2:**
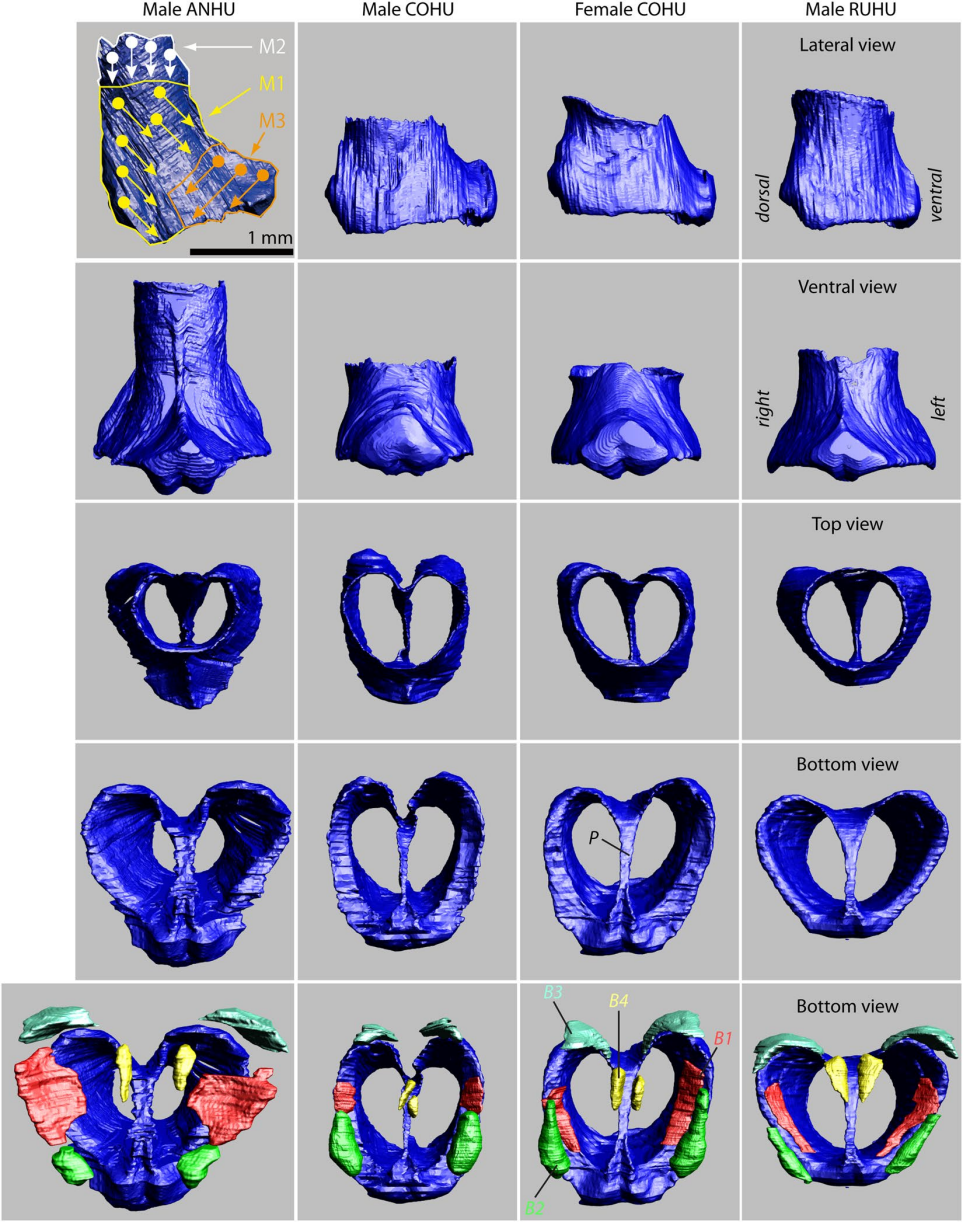
Three-dimensional models of the tympanum and four accessory cartilages from three hummingbird species reflecting qualitative differences in shape and size between the species. P, pessulus; B1 through B4 denote four accessory cartilages that are embedded in the lateral wall (B1–B3) and the MTM (B4). The large surface area of the tympanum serves as attachment area for intrinsic syringeal muscles. The arrows in the top left panel indicate attachment area and fiber orientation of three intrinsic muscles (M1, M2, M3). The accessory cartilages are in part inserted by those syringeal muscles and thereby help to posture and tension the vibrating tissue inside the syrinx.

This appears not to decrease the air space volume since bronchial length is much longer than in other small birds (Fig. 2). Expected tracheal length and diameter were calculated based on a model developed by Hinds & Calder^57^.

^A^Hinds and Calder^57^; ^B^Daley and Goller^67^; ^C^Suthers and Hector^42^; ^D^Suthers and Hector^43^.

#### Cartilages

**Figure 3:**
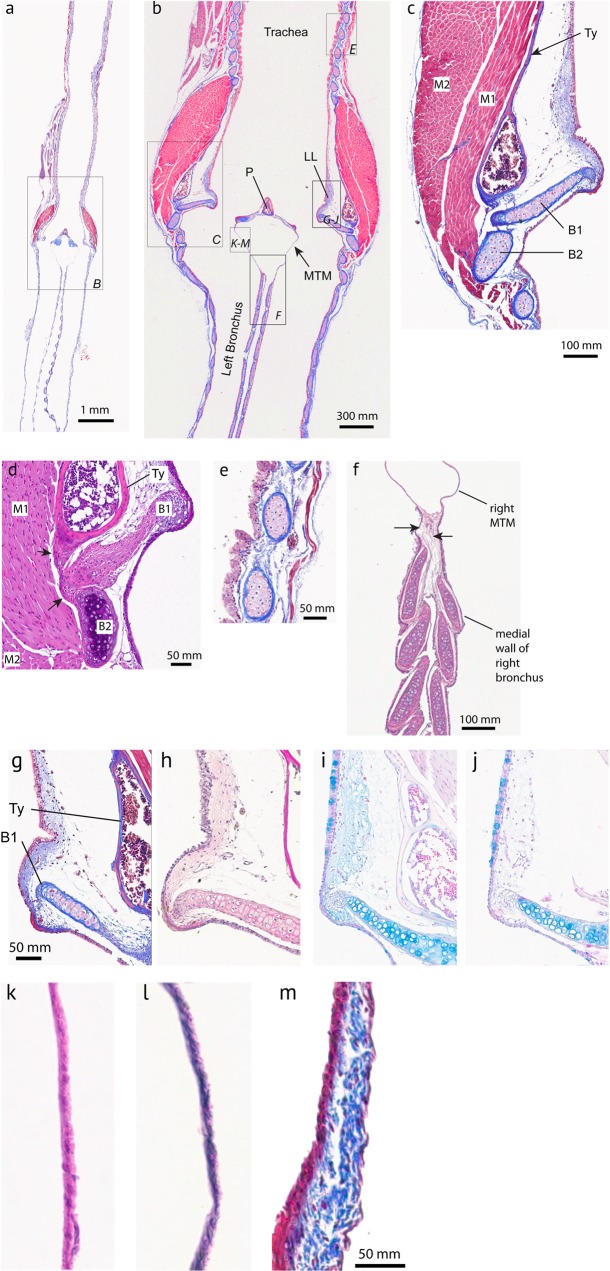
Histological sections of a hummingbird syrinx. Sections are coronal sections from three male Costa’s hummingbird syrinxes. (**a**) Overview of trachea, syrinx and two bronchi (trichrome stain). The square in (**a**) indicates the location of the higher magnification image in (**b**) and the squares in (**b**) indicate the location of the higher magnification images in (**c**–**m**). (**c**) B1 and B2 are accessory cartilages situated caudal from the tympanum (Ty). There are one or two expansions at the caudal end (**d**) and a single smaller expansion at the cranial end. Accessory cartilages (B1, B2, B3, B4) are positioned caudal to the tympanum. B1 occurs as a flattened structure that extends into the lumen, and at its lateral edge it is connected to the tympanum and to B2 by connective fibrous tissue (**d**, arrows). Note that B1 points almost horizontally into the lumen near the tracheobronchial junction, thereby seeming to obstruct airflow at this location. (**e**) Tracheal ring cross sectional area is oval-shaped at mid-organ level. (**f**) Bronchial half-rings were flat and long-shaped cross sections at mid-organ level. Arrows point to regions of high collagen density that connect the three cartilaginous structures. (**g**,**h**) The lateral labia consists of a single layer of cuboidal ciliated epithelium and a small amount of connective tissue below the epithelial layers. (collagen, blue stain in (**g**); no black elastin EVG stain in (**h**). (**i**,**j**) Hyaluronan (blue stain in (**i**), removal of hyaluronan by hyaluronidase digestion with subsequent AB staining (absent blue stain in (**j**)). (**k**–**m**) Medial tympaniform membrane (MTM) consists of two cellular layers (lumen side epithelium and airsac endothelium) with embedded elastic fibers (**l**). A thick layer of collagen fibers occurs near the dorsal end (**m**). Ty, tympanum; B1 and B2, first and second accessory cartilage; P, pessulus; LL, lateral labium; MTM, medial tympaniform membrane.

The caudal end of the trachea forms a robust tubular structure, the tympanum (Fig. 2). The tympanum refers to the fusion of lower tracheal rings forming a tube-like structure. The tympanum is mineralized with a density similar to bone (Fig. 3a,b). In contrast, trachea and bronchial cartilages consist of hyaline cartilage (Fig. 3e,f). Parts of the mineralized tympanum are hollow, for example the upper and lower rims filled with white and red blood cells as well as some connective tissue (Fig. 3c). The tympanum provides robust attachment points on the outside surface for the muscles of the syrinx.

Below the tympanum are four accessory cartilages (B1, B2, B3 and B4) that are likely involved in the biomechanics of syringeal valving movements during non-vocal breathing and during vocalization (Fig. 2, bottom row). B1 to B3 are located in the lateral wall close to the lateral-caudal edge of the tympanum: the B2 most dorsally, B3 most ventrally and B1 at the mid-organ level. The B1 cartilage is a flat plate-like structure. Interestingly in all specimens investigated, this largest of the accessory cartilages is oriented ventral to dorsally where it extends into the lumen of the tracheobronchial juncture in a position that would appear to obstruct the airflow. It is probable that this effect may develop *post mortem* or due to shrinking in association with the fixation process. The lateral base of the B1 cartilage is connected through thick connective fibers to both the caudal base of the tympanum and the B2 cartilage (Fig. 3D, arrows), suggesting that movements of B1 and B2 are tightly linked. Muscle fibers attach directly to the B2 (Fig. 3c,d) but we could not identify similar attachments to the B1 cartilage. The B3 cartilage is located ventral from the B1 element. The B4 is located in the MTM and lacks muscle attachments.

**Figure 4:**
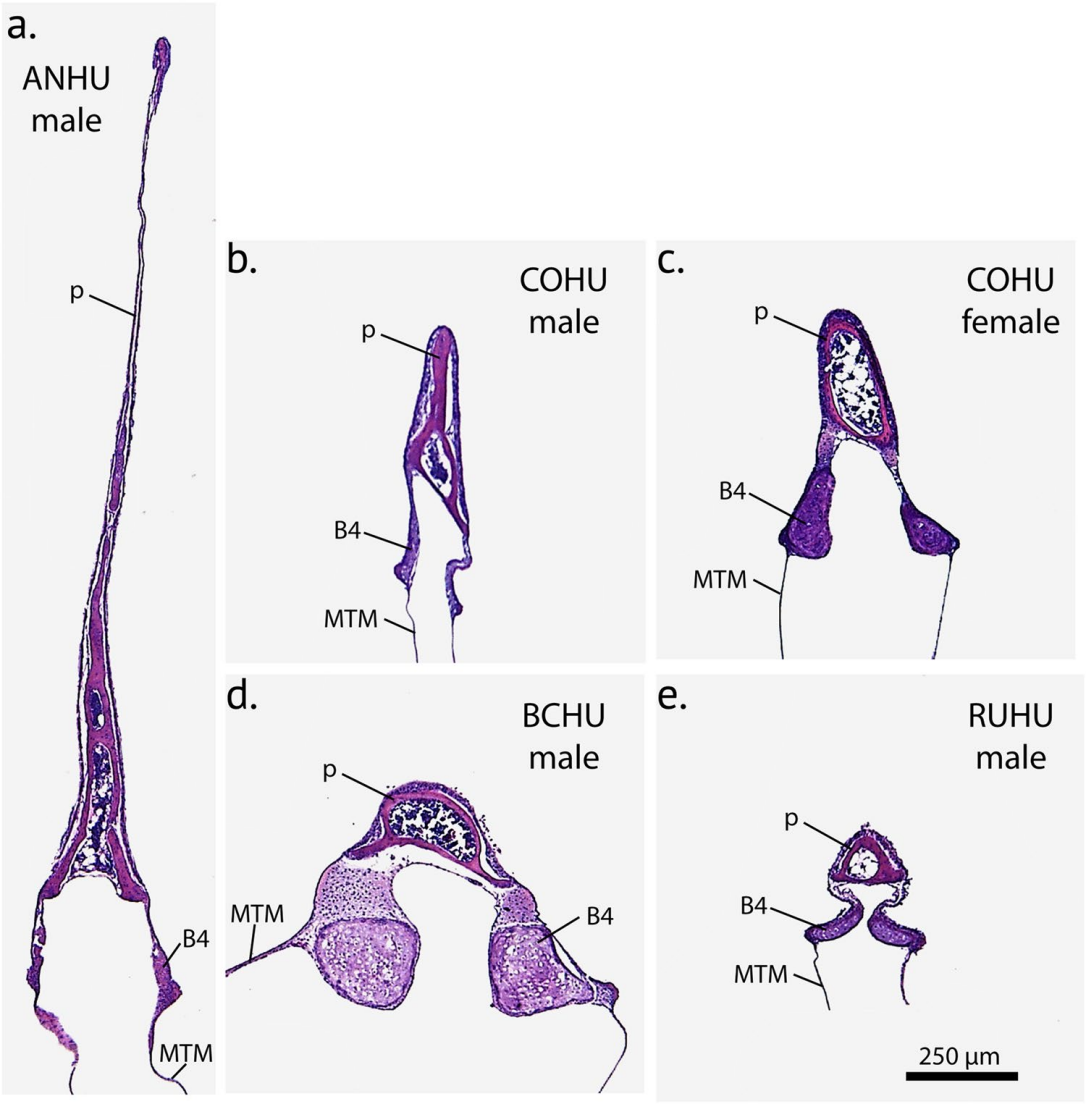
Coronal cross-sections of the tympanum at mid-organ level in four hummingbird species: (**a**) male Anna’s hummingbird, (**b**) male Costa’s hummingbird, (**c**) female Costa’s hummingbird, (**d**) male black-chinned hummingbird, (**e**) male rufus hummingbird. p = pessulus, B4 = fourth bronchial element, MTM = medial tympaniform membrane.

In hummingbirds the pessulus is a bony structure that has a hollow base that occurs at the juncture of the two bronchi and projects into the lumen of the tympanum, like that found in other avian species (Fig. 4a–e). There are considerable interspecies differences in the size and shape of the pessulus (see below). The left and right bronchi are connected through soft connective tissue in the cranial aspect (Fig. 3f). In many songbirds, a ligamentous connection is present here, however organized structure of a ligament was not observed in bee hummingbirds.

#### Musculature

**Figure 5:**
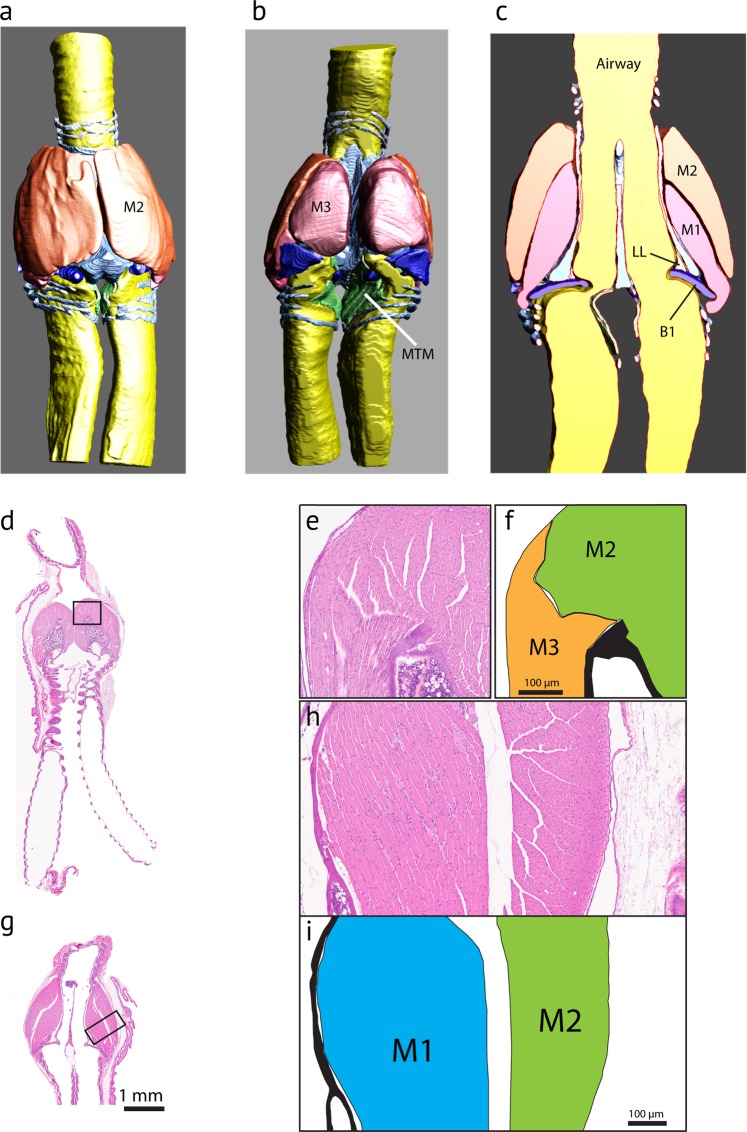
Three intrinsic muscles have been identified in the hummingbird syrinx. Ventral (**a**), dorsal (**b**) and mid-organ coronal (**c**) view of the segmented three dimensional surfaces of the syrinx of an adult male Anna’s hummingbird. The interactive pdf files corresponding to those images can be accessed via Supplementary Fig. 1. (**d**–**i**) Coronal cross sections of the syrinx (H & E stain) from dorsal (**d**–**f**) to ventral (**g**–**i**) of a male Anna’s hummingbird at 20 μm intervals. Two pairs of muscles move the syringeal cartilages. M1, M2, M3 – Muscles 1–3; B1–8, bronchial elements; T1–3, tracheal rings; Ty, tympanum; LL, lateral labium; MTM, medial tympaniform membrane.

Our goal was to understand anatomical arrangement of the hummingbird syrinx based on the connections of muscles to the tympanum and accessory cartilages. The following muscle fiber description was confirmed in 9 specimens investigated histologically and 4 specimens inspected by microCT imaging. Our investigation revealed three major intrinsic fiber groups (hereafter “muscle-1”, “muscle-2”, “muscle-3”). Figure 5a–c shows the 3D reconstruction of the three muscle pairs in an ANHU syrinx. The fibers of muscle-2 originate from the lateral and dorso-cranial surface of the tympanum, as well as from tracheal rings immediately cranial to the tympanum (Figs. 3 and 5). They insert on the ventro-lateral edge of the B2 element (Fig. 3d). The fibers of muscle-1 insert on the surface of the lateral tympanum slightly below muscle-2, and then insert on the dorsal end of the B2 element (Figs. 3b and 5c–i). A third group of fibers (muscle-3) originates at the medial raphe and inserts on the B3 element. Attachment of the three muscles on the tympanum is indicated in Fig. 3 (top left panel).

Also present are paired *tracheolateral* muscles that attach extrinsically to the syrinx and extend rostrally as a thin layer to attachment points on the lateral trachea. Absent are equivalents to the extrinsic *sternotracheal* muscles that connect the tracheal ring T1 to the sternum in other avian lineages.

#### Soft vibrating tissue

There are laterally-positioned accumulations of soft tissue mass (hereafter ‘lateral labia’) within the airway rostral to bronchial cartilage B1. The lateral labia are composed of lamina propria and a thin layer of cuboid epithelium with cilia (Fig. 3g–j). The lamina propria is composed of a ~20 µm thin layer of protein fibers, containing primarily collagen (Fig. 3g,h). Elastin fibers were not found (Fig. 3h). The lateral labia contain also hyaluronan (Fig. 3i,j), an interstitial space substance with critical importance for viscoelastic properties for vibrating tissue.

Lamina propria of the lateral labium contains collagen (blue stain in Fig. 3g) but no elastin (no black coloration in EVG stain in Fig. 3h). It also contains hyaluronan (blue stain in Fig. 3i). The blue stain in the lamina propria is gone after the removal of hyaluronan by hyaluronidase digestion with subsequent AB staining (Fig. 3j). Goblet cells, located in the ciliated epithelium of the lateral labium, retain their blue coloration after the hyaluronidase digestion and subsequent Alcian blue stain.

In contrast to the passerine syrinx, no structures resembling medial labia were found but MTMs were present. The MTMs were connected to each other through soft tissue in the caudal aspect (Fig. 3f) and consisted of a single cell epithelial layer on the luminal side and a second layer of airsac epithelium (Fig. 3k–m). A few elastic fibers are embedded between the two layers at mid-organ level (Fig. 3l) but more collagen is embedded between the two cellular layers (Fig. 3m). The pessulus in ANHU and COHU reaches far rostral (Fig. 4) and thereby separates the left and right set of soft tissue masses (lateral labium and MTM). It is therefore reasonable to assume that both sets operate independently and constitute two sound sources.

#### Species level differences

Inspection of the 3-D models of our hummingbird species suggested species level differences. For example, the syrinx of *Calypte* spp. showed the largest muscle volumes and the tympanum in ANHUs was longer than in the other species. In ANHUs, the pessulus forms a large septum that clearly separates the lumen of the tympanum into two cavities (Fig. 4a). The consequence is that the vocal organ is separated into left and right sound sources in this species. The COHU also has a slightly elongate pessulus that is intermediate in length and which only extends slightly into the tympanic cavity resulting in a mostly open lumen (Fig. 4b,c). In contrast, the BCHU and the RUHU have a much-reduced triangle-shaped pessulus with a wide base (Fig. 4d,e), resulting in a single tympanic chamber.

**Figure 6:**
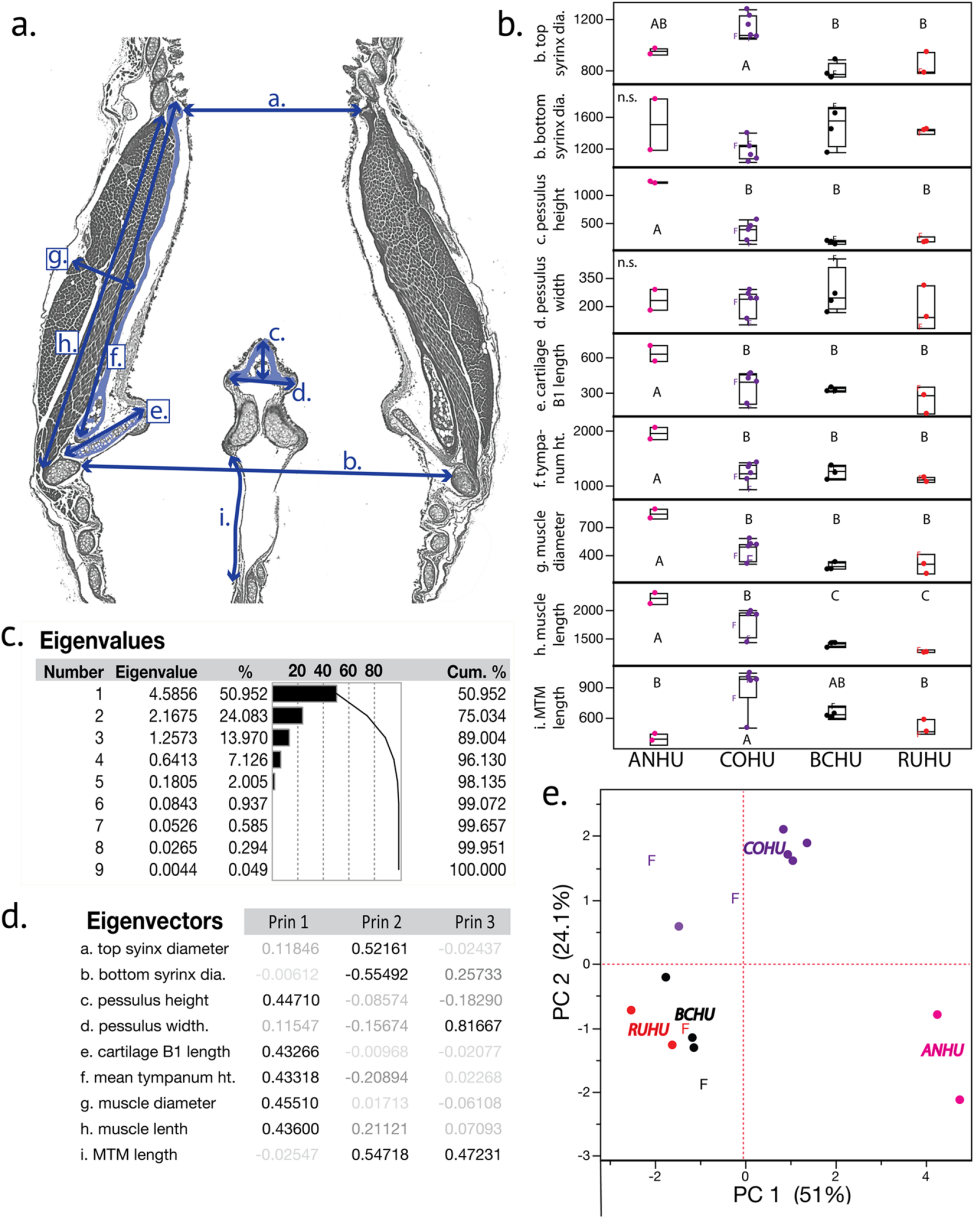
Species-level analysis of hummingbird syrinx morphology (n = 18 birds). (**a**) Schematic of the syrinx measurements used in a representative serial section, for (**a**) top syrinx diameter, (**b**) bottom syrinx diameter, (**c**) pessulus height, (**d**) pessulus width, (**e**) length of cartilage B1, (**f**) mean tympanum height, (**g**) mean muscle diameter, (**h**) mean muscle length and (**i**) is the MTM length (the only curvilinear measurement). (**b**) Individual character measurements for each species, where different letters represent honestly significant differences from post-hoc Tukey tests for each trait independently. Quartile differences are represented by box-whisker plots and female samples are denoted by ‘F’. Y-axes are in μm. (**c**) Eigenvalues from a correlation-based PCA of the 9 characters in this analysis. (**d**) Eigenvectors with heavily loading characters represented as bold text within each vector. (**e**) Biplot of PC 1 versus PC 2. Species are represented by different colors clustered around the abbreviation for each species, and females are represented by ‘F’.

In the dataset containing the nine morphological syrinx measurements made in the frontal plane (Fig. 6a), we found a strong species-level effect (MANOVA, Wilk’s λ_9, 27_ = 7.1 × 10^−4^, p = 0.0024). Seven of the nine characters showed significant species-level differences, based on Tukey HSD *post hoc* tests evaluated for each character individually (Fig. 6b). Only the bottom syrinx diameter and the pessulus width showed no differences across species. Of the four bilateral measurements only tympanum height showed asymmetry in that the left side was usually longer than the right side (paired t-test, 2-tailed, t = 2.7, df = 12, p = 0.02). Muscle length, muscle diameter and B1 length did not show lateral differences, however it is not clear whether more subtle differences are undetectable with our technique or if the blocking techniques used for these tissues are responsible for these observations. A PCA reduction of these data based on the correlation matrix revealed the first three eigenvalues to contain ~89% of the overall variation, with PC 1 containing ~51% of the variation and describing the pessulus height, cartilage B1 length, the tympanum height as well as the muscle length and diameters (Fig. 6c,d). PC 2 was composed of ~24% of the variation, and described the tracheal and bronchial diameters, as well as the MTM length. PC 3 amounted to ~14% of the variation in the dataset and was composed of the pessulus width and MTM length. A biplot of PC 1 versus PC 2 showed a clear species-level separation (Fig. 6e) with positive loadings of ANHUs along PC 1 axis reflecting the larger size of the syrinx in that species, particularly in the measurements along the longitudinal axis and in muscle dimensions. COHUs were intermediate in these traits between ANHU and the BCHU/RUHU, particularly in the muscle measurements. The positive loadings in PC 2 in COHUs reflected a wider tracheal opening and longer MTM length in that species (Fig. 6b), suggesting a distinct shape difference in syrinx morphology in the COHU compared to the other three species (Fig. 6b). The BCHUs and RUHUs, despite representing separate lineages, showed no significant differences in any of the measured traits on a pair-wise basis, and in the PCA points clustered close to one another with some evidence of a small separation between species. We were able to represent a few specimens from females that are denoted as ‘F’ in Fig. 6e. Two female COHUs were present in the study and both seemed to show smaller muscle measurements than four of the five males of the species, suggesting that there may be sexual dimorphism in this trait.

### Vocal characteristics and heliox experimentation

In captivity we recorded four distinct call types (Supplementary Fig. 2a–d), two of which were also recorded under conditions of heliox. *Chip calls* were frequently produced in all three captive ANHUs in response to mild and moderate disturbances and are previously described for this species in a range of behavioral contexts^40,41^. F_0_ of chip calls did not increase in heliox (maximum F_0_ in air = 8.6 ± 0.3 kHz, in heliox = 8.5 ± 0.3 kHz) and was much lower than predicted for an aerodynamic whistle in the presence of helium (12.8 ± 0.3 kHz; paired t-test, t = −45, N = 3, p > 0.001) (Supplementary Fig. 2e). Call duration was not different (paired t-test, t = 1.9, N = 3, p = 0.1). *Phee calls* (Supplementary Fig. 2b) from a single individual were recorded in normal air and heliox. Phee calls recorded in heliox were not higher in fundamental frequency (Supplementary Table 2; F_0_ in air and in heliox = 9.7 ± 0.2 kHz, N = 20 calls in each condition) and fundamental frequency was also lower than predicted (4.8 ± 0.3 kHz) for a whistle in the presence of heliox (Supplementary Fig. 2e). We also note the presence of *screach* and *warble* calls as being distinct from the previous two (Supplementary Fig. 2c,d), however these are not included in our analysis because these vocalizations were not recorded in heliox. In sum, the results indicate that sound is produced by an airflow-induced tissue vibration mechanism.

Vocal tract filter properties depend on the density of the gas in the vocal tract. Next, we tested whether different filter properties affected the amplitude of the second harmonic frequency (2F_0_) in normal air and in heliox relative to F_0_ at peak fundamental frequency. Results were inconsistent. In two animals, amplitude of 2F_0_ relative to F_0_ was higher in heliox than in normal air, in one animal the result was reversed (Supplementary Table 2).

## Discussion

The syrinx of four bee hummingbird species is characterized by three pairs of intrinsic muscles, a large tympanum which serves as an attachment for those muscles, and four accessory cartilages are involved in syringeal movements. This syrinx morphology sets hummingbirds apart from other groups in the larger *Strisores* clade, including *Caprimulgiformes*^13,42^ and other *Apodiformes*^43^. The order Apodiformes contains besides hummingbirds, only treeswifts (Hemiprocnidae) and swifts (Apodidae) as recent groups. Recent investigations of glossy swiftlets (*Collocalia esculenta*) and Australian swiftlets (*Aerodramus terraereginae)* confirm a syrinx design without intrinsic musculature, without accessory cartilages, and without a boney or cartilaginous structure resembling a tympanum, but with an avian-typical intra-thoracic position^44,45^. Thus, multiple anatomical features distinguish the hummingbird syrinx from that of the other Apodiformes.

Beddard^13^ and more recent work by Zusi^17^ have noted that the hummingbird syrinx resembles the Passerine vocal organ based on the appearance of external musculature of specimens that they examined. We confirm the similarity of the syrinx based on the combination of four features that passerines and at least bee hummingbirds share: (a) multiple sets of intrinsic muscles, (b) a large tympanum, (c) the presence of multiple accessory cartilages and (d) the presence of two sets of opposing soft tissue masses. Our investigation expands on previous notions by specifying the number of intrinsic muscles and their predominant fiber orientation. The intrinsic muscles insert on the large surface of the tympanum and exert force on a set of four accessory cartilages and at least one bronchial half-ring in each sound source. We also establish that the hummingbird syrinx, like in passerines, consists of two sets of lateral labium and MTM, one set located in each proximal bronchus which is a prerequisite for the existence of a bipartite syrinx, i.e. two sound sources in a single vocal organ.

The description of muscle fiber orientation provides insights into the biomechanics and movement of the hummingbird syrinx which should allow for the development of testable hypotheses. The hummingbird syrinx is a bipartite syrinx, i.e. two sets of lateral labia and medial tympaniform membranes are separated one in each bronchus. Syringeal movements position the soft tissues in those two sound sources. Based on the histological appearance of near-perpendicular fiber orientations of muscle-2 and muscle-3, as well as on the different insertion points, we speculate that the activity of three muscles have opposing effects, i.e. adduction or abduction of the syrinx. Muscle-1 mostly tenses the external labium, and subsequently increases or decreases vibration rate and sound fundamental frequency. These muscles likely have functional equivalents to passerine syrinx musculature. A summary of insertion characteristics of the hummingbird syrinx musculature and a comparison with the passerine syrinx are provided in Table 2.

**Table 2:** Three main muscles were identified in the hummingbird syrinx based on fiber orientation and insertion points.

	Origin	Insertion	Proposed function	Passerine equivalent
Muscle 1	Lateral surface of the tympanum	Bronchio-syringeal cartilage B2	Direct rotation of B2 and indirect movement of B1 leading to an increase in tension of lateral labium	M. syringealis ventralis → Main regulator of fundamental frequency
Muscle 2	Lateral surface of the tympanum and tracheal rings immediately cranial to the tympanum	Bronchio-syringeal cartilage B2	Abduction of lateral labium	M. tracheobronchialis → abduction of lateral labium
Muscle 3	Medial raphe	Bronchio-syringeal cartilage B3	Adduction of lateral labium	M. syringealis dorsalis → adduction of lateral labium

The labels ‘Muscle 1’, ‘Muscle 2’ and ‘Muscle 3’ are used for convenience but should not imply three separate muscles. The comparison with the passerine syrinx is based on data presented by Goller and Suthers^22,23^. Bronchio-syringeal cartilages B1 - B4 are often referred to as *accessory cartilages*^21^.

The presence of three major pairs of intrinsic syrinx muscles in bee hummingbirds, which allow posturing of vocal vibrating tissue, is one prerequisite for increased pitch control^46^. Their interaction helps to (a) posture (i.e. abduct and adduct) lateral labia and the MTM and (b) to control the tension of the vibrating tissue. Considering the enormous fundamental frequency range (1.5 to 10 kHz; i.e. two to three octaves) that hummingbird vocalizations cover^4–6,10,11^, it is very likely that the proposed function for muscle 1, i.e. modify labia tension (Table 2), bears some similarity to an equivalent muscle in the oscine syrinx. In contrast, the biomechanical mechanism to position lateral labia and MTM in swiftlets to produce echolocation sounds is entirely facilitated by two tracheal muscles, the tracheolateralis and the sternotrachealis^43^, the latter of which is not found in bee hummingbirds.

In all four hummingbird species we found three lateral accessory cartilages located in the lateral bronchial wall and a fourth accessory cartilage embedded in the rostral MTM at the base of the pessulus. Intrinsic syringeal muscles 1 through 3 attach to the accessory cartilages. A review of the literature suggests that the only other group in which accessory cartilages have been described in the syrinx are Passeriformes^13,21,28,47,48^. The exact function of accessory cartilages in the syrinx is unknown but it seems safe to speculate that they serve a similar function as other sesamoid bones in the body. They act like pulleys and/or provide a smooth surface for tendons to slide over or to attach to and thereby enhance the ability to transmit controlled muscular forces in structurally complex organs.

Airflow drives vibrations of lateral labia and MTM. Our heliox experiments suggest that vocalizations are produced when soft tissue structures are set into vibration by an airflow. Fundamental frequency consistently did not change in heliox. The results for two call types resemble findings in previously tested bird species in a heliox atmosphere^49–51^. The composition of the extracellular matrix of those vibrating structures ensures that the tissue is pliable enough and can be drawn into vibration^46^. The mechanical properties of those vibrating structures determine their vibration rate, i.e. fundamental frequency. We found that the lamina propria of the lateral labia and the MTM were composed predominantly of collagen fibers and hyaluronic acid, however, few elastic fibers were found. This is in line with findings in songbirds where species with the greatest frequency range were found to have a more collagen-rich soft tissue organized in a layered structure compared to those with low frequency ranges^25^.

A third variable of fundamental frequency control is the subsyringeal pressure. The placement of the syrinx outside the thoracic cavity presents an interesting problem in the application of subsyringeal pressure. In the typical avian intrathoracic arrangement, pressure changes in the interclavicular airsacs surrounding the syrinx have effects on frequency modulation of vocalizations^52^. In hummingbirds, the interclavicular airsac extends into the neck region where a portion maintains a connection to the syrinx, and where it may potentially exert an effect on sound production^17^. However, the degree to which pressure gradients may be transmitted into the neck to exert their effects on the hummingbird syrinx has not been determined. The mechanisms of regulating syringeal pressure in the hummingbird are thus important directions for future research.

Finally, a fourth critical variable is the vocal tract resonance. A sound source that is phonated with a fundamental frequency near the resonance of the downstream vocal tract filter can benefit by receiving a boost in transfer efficiency, and thereby an increased sound amplitude that is radiated from the mouth. This is known for songbirds^53,54^ and some singing styles in humans^55^ and is suggested to be an important factor in the origin of the avian syrinx^56^. Due to the intrathoracic position of the syrinx in the respiratory tract of most birds, their vocal tract is much longer than that of other similar sized vertebrates ^56^ which can enhance vocal tract resonance. However, hummingbirds are an exception, having a comparatively short trachea of approximately 9 mm (Fig. 1b, Table 1). A model for tracheal length presented by Hinds and Calder^57^ predicts a tracheal length of 18 mm for a bird the size of hummingbirds to efficiently boost sound at the fundamental frequency near 4.8 kHz. In contrast, a 9 mm trachea of hummingbirds is only half the expected length for a ~4 g bird (Table 1), which would instead efficiently support higher frequencies around 9.7 kHz (i.e., the first resonance of a 9 mm long uniform tube). In order to operate the syringeal labia near 9.7 kHz, in theory, large forces need to be generated to sufficiently tense the labia so that high vibration rates can be achieved^58,59^. It is tempting to speculate that the syringeal displacement into the neck and the associated acoustic shortening of the trachea have subsequently contributed to the evolution of a highly muscular syrinx that is able to modulate fundamental frequency over a two to three octave range. The only other recorded example of an extrathoracic syrinx is that of the roseate spoonbill (*Platalea ajaja*)^60,61^.

Previously reported similarities between neuro-anatomical structures of vocal learning hummingbirds and Passeriformes^7,62^ are paralleled by similarities in the peripheral vocal organ morphology. We note that both male BCHU and RUHU, which phylogenetic analysis suggest have both separately lost the ability to produce learned song and instead use feather sounds^2^, have comparatively small syrinxes that are relatively similar to one another in their morphology. In contrast, the two vocal learning hummingbirds, ANHU and COHU, have larger and more morphologically divergent syrinxes. The differences in syrinx morphology among the four species we examine here suggests that vocal learning is related to syrinx morphology in hummingbirds. Interestingly the resemblance in syringeal morphology that occurs in the hummingbirds and oscines does not extend to the third group of vocal learning birds, the parrots (Order: Psittaciformes)^7,62–64^. The tracheal parrot syrinx contains only a single set of vibrating membranes in the lateral wall of the lower trachea^21,65^ and the cartilaginous framework contains a few fused bronchial half rings at its caudal end, but nothing that resembles a tympanum^21^. Furthermore, the less complex musculature is mainly comprised of an intrinsic *syringealis superficialis* muscle that controls abduction and adduction, and a second intrinsic *syringealis profundus* muscle whose primary role is to regulate membrane tension^20,66^. In sum, all three major groups of vocal-learning birds share the ability to regulate abduction and adduction as well as tension, but it appears that only the distantly-related songbirds and hummingbirds have converged on a similar vocal organ morphology, while parrots employ a very different cartilaginous framework and biomechanic approach to facilitate a similar airflow-induced vocal fold vibration. Thus, a seemingly radical adaptation such as vocal learning with associated fundamental changes in forebrain neurological structures^8,62^ is not necessarily associated with equally radical peripheral adaptations.

## Supplementary information

Supplementary File 1.

Supplementary Information
