# Supplementary figures and images for: The vocal organ of hummingbirds shows convergence with songbirds
Source: Sci Rep. 2020 Feb 6;10:2007. doi: 10.1038/s41598-020-58843-5 (PMC7005288; doi:10.1038/s41598-020-58843-5)

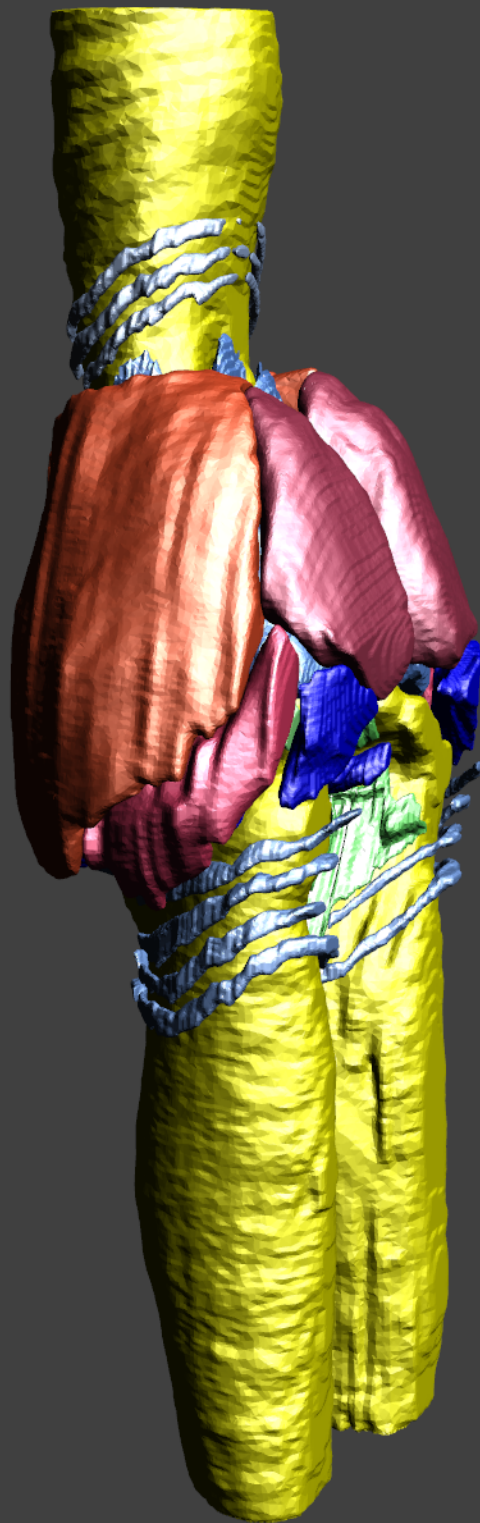

Supplement: Supplementary file 1 [file 41598_2020_58843_MOESM1_ESM.pdf]
