# Supplementary material for: The vocal organ of hummingbirds shows convergence with songbirds
Source: Sci Rep. 2020 Feb 6;10:2007. doi: 10.1038/s41598-020-58843-5 (PMC7005288; doi:10.1038/s41598-020-58843-5)
Supplement: Supplementary file 2 — Supplementary Information [file 41598_2020_58843_MOESM2_ESM.pdf]

Supplementary Information

The vocal organ of hummingbirds shows convergence with songbirds

Tobias Riede and Christopher R. Olson

Department of Physiology
College of Graduate Studies
Midwestern University,
19555 N 59th Ave
Glendale, AZ, USA 85308

Supplementary Table 1
Supplementary Table 2
Supplementary Figure 1 (see attached interactive PDF file)
Supplementary Figure 2

Supplementary Table 1. Hummingbirds used in this study. Body masses taken from Dunning (2008). The ability to produce song is indicated in those that are known to have learned song and/or the presence of forebrain vocal nuclei. Sample sizes include total numbers per species/sex with the number of juvenile birds in that category indicated in parentheses.

Species	Abbreviation	Scientific name	Sex	Body mass (g)	N
Anna's	ANHU	*Calypte anna*	Male	4.5	2(0)
Costa's	COHU	*Calypte costae*	Male	3.0	6(2)
Costa's	COHU	*Calypte costae*	Female	3.2	2(1)
Black-chinned	BCHU	*Archilochus alexandri*	Male	3.2	4(0)
Black-chinned	BCHU	*Archilochus alexandri*	Female	3.6	1(0)
Rufous	RUHU	*Selophorus rufus*	Male	3.5	2(2)
Rufous	RUHU	*Selophorus rufus*	Female	3.5	1(1)

Supplementary Table 2. Fundamental frequency (maximum and minimum), call duration (Dur) and dB levels of the second harmonic ($2F_0$) relative to F_0 in normal air and in heliox.

	$F0_{max}$ (kHz) in air	$F0_{max}$ (kHz) in heliox	$F0_{min}$ (kHz) in air	$F0_{min}$ (kHz) in heliox	Dur (ms) in air	Dur (ms) in heliox	$2F_0$ (dB) in air	$2F_0$ (dB) in heliox
Bird 1	8.8 ± 1.0	8.8 ± 0.3	2.3 ± 0.5	2.8 ± 0.6	11.1 ± 1.6	10.1 ± 1.1	18.8 ± 6.5	12.3 ± 4.0
Bird 2	8.2 ± 0.3	8.1 ± 0.3	2.5 ± 0.4	2.3 ± 0.5	7.9 ± 1.1	7.9 ± 1.1	11.6 ± 5.7	14.9 ± 3.0
Bird 3	8.7 ± 0.3	8.6 ± 0.2	2.1 ± 0.3	2.3 ± 0.5	17.7 ± 1.6	17.0 ± 1.7	16.4 ± 4.3	9.9 ± 4.1
Bird 3, phee call	9.7 ± 0.2	9.7 ± 0.2			38.2 ± 11.6	36.7 ± 7.6	19.4 ± 4.4	19.5 ± 8.2

### See Supplementary File 1 to view figure ###

Supplementary Figure 1. Interactive PDF file of an adult male Anna's Hummingbird Syrinx in three dimensions. Viewer may zoom in, rotate and hide selected components to inspect the structure. Anatomical dead space: yellow; muscle fascicles: red; accessory cartilages: dark blue; tympanum, bronchial and tracheal rings: light blue; medial tympaniform membranes and lateral labia: green. Figure is best viewed using Adobe Acrobat Pro (ver. 9).

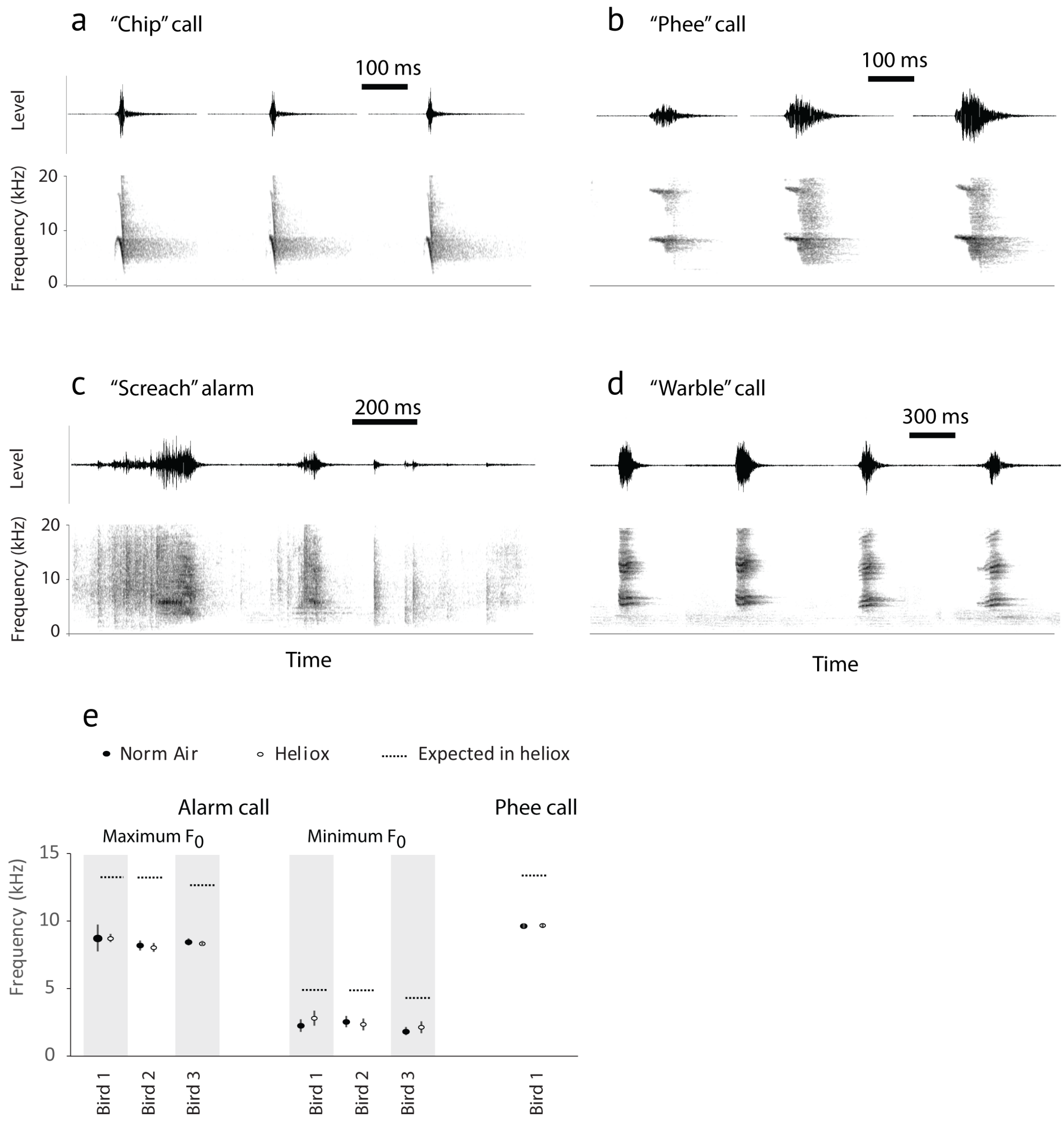

Supplementary Figure 2: Four different call types were produced by captive male Anna's Hummingbirds (n = 3). **a-d:** calls are depicted as waveforms (top) and spectrograms (bottom). **e:** Acoustic properties of Chip calls and of Phee calls produced in normal air and heliox. Maximum fundamental frequency (F_0) of calls in air and heliox (mean±s.d.). Fundamental frequency remained below the predicted frequency estimated from the frequency response of a whistle. Horizontal dashed lines depict expected F_0 in heliox.
